# Towards Novel 3-Aminopyrazinamide-Based Prolyl-tRNA Synthetase Inhibitors: In Silico Modelling, Thermal Shift Assay and Structural Studies

**DOI:** 10.3390/ijms22157793

**Published:** 2021-07-21

**Authors:** Luping Pang, Stephen D. Weeks, Martin Juhás, Sergei V. Strelkov, Jan Zitko, Arthur Van Aerschot

**Affiliations:** 1 Biocrystallography, Department of Pharmaceutical and Pharmacological Sciences, KU Leuven, Herestraat 49—Box 822, 3000 Leuven, Belgium; luping.pang@kuleuven.be (L.P.); sweeks@pledge-tx.com (S.D.W.); sergei.strelkov@kuleuven.be (S.V.S.); 2 Medicinal Chemistry, Rega Institute for Medical Research, KU Leuven, Herestraat 49—Box 1041, 3000 Leuven, Belgium; 3 Pledge Therapeutics, Gaston Geenslaan 1, 3001 Leuven, Belgium; 4 Faculty of Pharmacy in Hradec Králové, Charles University, Akademika Heyrovského 1203, 500 05 Hradec Králové, Czech Republic; juhasm@faf.cuni.cz

**Keywords:** prolyl-tRNA synthetase, inhibitor, thermal shift assay, X-ray crystallographic studies, in silico modelling

## Abstract

Human cytosolic prolyl-tRNA synthetase (HcProRS) catalyses the formation of the prolyl-tRNA^Pro^, playing an important role in protein synthesis. Inhibition of HcProRS activity has been shown to have potential benefits in the treatment of fibrosis, autoimmune diseases and cancer. Recently, potent pyrazinamide-based inhibitors were identified by a high-throughput screening (HTS) method, but no further elaboration was reported. The pyrazinamide core is a bioactive fragment found in numerous clinically validated drugs and has been subjected to various modifications. Therefore, we applied a virtual screening protocol to our in-house library of pyrazinamide-containing small molecules, searching for potential novel HcProRS inhibitors. We identified a series of 3-benzylaminopyrazine-2-carboxamide derivatives as positive hits. Five of them were confirmed by a thermal shift assay (TSA) with the best compounds **3b** and **3c** showing EC_50_ values of 3.77 and 7.34 µM, respectively, in the presence of 1 mM of proline (Pro) and 3.45 µM enzyme concentration. Co-crystal structures of HcProRS in complex with these compounds and Pro confirmed the initial docking studies and show how the Pro facilitates binding of the ligands that compete with ATP substrate. Modelling **3b** into other human class II aminoacyl-tRNA synthetases (aaRSs) indicated that the subtle differences in the ATP binding site of these enzymes likely contribute to its potential selective binding of HcProRS. Taken together, this study successfully identified novel HcProRS binders from our anti-tuberculosis in-house compound library, displaying opportunities for repurposing old drug candidates for new applications such as therapeutics in HcProRS-related diseases.

## 1. Introduction

Aminoacyl-tRNA synthetases (aaRSs) are a class of essential enzymes found in all cells. They are responsible for catalysing the ligation of an amino acid to its cognate tRNA in an ATP-dependent manner [1]. The charged tRNAs are subsequently used for protein synthesis at the ribosome. To date, there are 36 known aaRSs encoded by distinct genes present in human cells. Among them, 16 are found in the cytoplasm, 17 are transported and exclusively localized in the mitochondria and the remaining three (GlnRS, LysRS and GlyRS) show dual localization [2]. Many human diseases are associated with aaRS dysfunction, such as the overexpression and the enhancement of aaRS catalytic activity in some cancers [3] and the emergence of clinically relevant aaRS mutants in genetic diseases [4,5,6]. In addition to their important roles in protein synthesis, aaRSs have also been shown to have certain non-canonical functions [7] being involved in various physiological and pathological processes, including post-translational modifications, autoimmune diseases, angiogenesis, fibrosis and neuropathy [4]. Therefore, aaRSs are attractive targets in the development of therapeutic agents against multiple human diseases [8,9].

**Figure 1:**
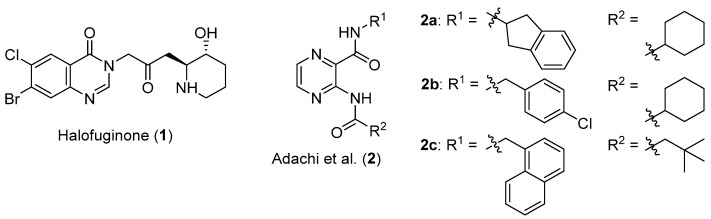
Chemical structures of halofuginone (**1**) [10] and confirmed HcProRS inhibitors **2a**, **2b**, **2c** [16].

Notably, glutamyl-prolyl-tRNA synthetase (EPRS) is a dual function enzyme that catalyses the formation of charged glutamyl-tRNA and prolyl-tRNA in the cytoplasm. The human ProRS (HcProRS) activity is located at the C-terminal region of this protein and inhibition of this enzyme has been recognized as a promising approach for treatment of HcProRS-related diseases. Halofuginone (Figure 1), a validated ProRS inhibitor, occupies both the proline (Pro) binding site and the 3′-end of cognate tRNA binding site in an ATP-dependent manner [10]. Halofuginone has shown to have excellent anti-malaria, anti-fibrosis and anti-cancer activities [10,11,12,13,14]. However, due to its poor selectivity between species, further clinical application has been severely hampered [15]. Recently, Adachi et al. identified a number of pyrazinamide-based HcProRS inhibitors (Figure 1, compounds **2a–c**) via a high-throughput screening (HTS) system based on a bioluminescent enzymatic assay. Among them, the best compound **2a** demonstrated potent binding affinity with a K_d_ of 0.76 nM for HcProRS in the presence of Pro [16]. Biochemical data complemented with a crystal structure further proved that this compound is an ATP-competitive inhibitor that works in a Pro-dependent fashion.

Pyrazinamide (PZA) is a clinically validated first-line antitubercular drug for the clinical treatment of active *Mycobacterium tuberculosis* infections and has also been considered as a bioactive chemical scaffold used for various chemical modifications [17,18,19,20,21,22]. Zitko’s group reported a large series of pyrazinamide derivatives as part of efforts for the development of new antimicrobials, especially as antimycobacterial drugs [17,18]. The in-house library contains a series of 3-substituted-*N*-benzylpyrazine-2-carboxamide derivatives, which share high structural similarity with confirmed HcProRS inhibitor **2a**. This motivated us to systematically investigate the potential of these compounds as HcProRS inhibitors. We therefore performed molecular docking to virtually screen our small molecule library using the available compound **2a**-bound HcProRS structure (PDB ID: 5VAD) as a starting model. Positive hits were further confirmed by thermal shift assay (TSA) and co-crystal structures. These results enabled us to identify five lead compounds which would be useful for future structure-based drug design of more potent HcProRS inhibitors.

## 2. Results

### 2.1. Docking Compound Library with HcProRS Ligand-Bound Structure

**Figure 2:**
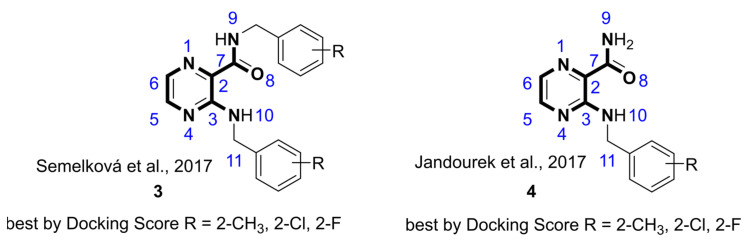
Selected hits from the initial in silico screening of our in-house database [17,18]. The atom numbering of the compound scaffold is shown in the structure.

Using the ternary complex of HcProRS:Pro:**2a** (PDB ID: 5VAD) as a starting model, we performed in silico screening based on molecular docking of 2,3-disubstituted pyrazines from our in-house library (97 compounds in total; for the experimental procedure, see Supplementary Materials). The results indicated that the highest scoring derivatives were 3-benzylamino-*N*-benzylpyrazine-2-carboxamides (Figure 2, general formula **3**), originally published as antimycobacterial compounds [18]. Some of our derivatives showed the same binding mode compared to the confirmed inhibitor **2a**, exerting all H-bond interactions except for the H-bond to the carbonyl oxygen at C3-amine position, which is missing in our structures (Figure 2 and Figure S1). The RMSD calculated for the shared subset of atoms (bolded in Figure 2), taking the crystallographic pose of **2a** as a reference, ranged from 0.42 Å (minimum value) for derivatives with a substitution in position 2 of the benzyl ring up to 0.86 Å (maximum value) for derivatives (**3**) with substituents in position 4 of the benzyl ring (R = 4-CF_3_, 4-OCH_3_, 4-Cl, 4-CH_3_, 3,4-diCl). The poses of these 4-substituted derivatives demonstrated worse docking scores and, due to the distortion of the carboxamide moiety attached at the C2 position of pyrazine, also lacked the H-bond between the NH of the carboxamide group and the backbone of Thr1164. Both in the confirmed inhibitor **2a** (crystallographic structure) and in our compounds of general structure **3**, the substituent on the C2-carboxamide group exerts only non-specific hydrophobic interactions to the enzyme. For this reason, we also focused on our in-house derivatives of general structure **4** with an unsubstituted C2-carboxamide group [17]. For these, we generally observed worse docking scores (as expected because of the lower number of heavy atoms due to the lack of the substituent on the C2-carboxamide) and more flexibility in terms of different binding modes. In most cases, compounds **4** with substitution in position 4 of the benzyl ring were not able to reach the binding mode of the confirmed inhibitor.

To sum up, the initial in silico screening suggested that both compounds of general structure **3** and **4** are capable of binding to HcProRS in the same mode as the confirmed inhibitor **2a**. The docking also showed preference for derivatives with substitution in position 2 of the benzyl ring as indicated by both docking scores and RMSD values. On the contrary, 4-substituted derivatives were discouraged by molecular docking, except for sterically small R = 4-F. The docking scores and RMSD values of selected derivatives are present in Table 1, while full results are present in the Supplementary Materials Table S1.

### 2.2. Confirmation of the Hits from Virtual Screening by a Thermal Shift Assay

**Figure 3:**
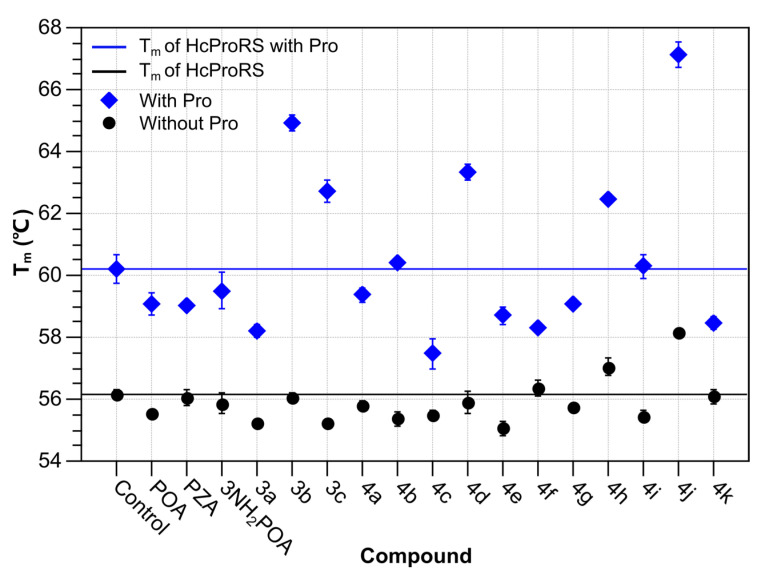
Evaluation of compound binding by a fluorescence-based thermal shift assay. T_m_ values of HcProRS were measured by incubating with 100 µM of compound in the absence (black circles) or presence (blue diamonds) of 1 mM Pro. The control samples of the two separate experiments correspond to the T_m_ of HcProRS with vehicle in the absence or presence of 1 mM Pro, respectively. The black and blue lines correspond to the T_m_ values of the respective control samples. Each measurement was performed in triplicate with standard errors shown.

Selected compounds from in silico screening (general structures **3** and **4**, both active and inactive as predicted by docking) along with relevant fragments (pyrazinoic acid, POA; pyrazinamide, PZA; and 3-aminopyrazinoic acid, 3-NH_2_-POA) were evaluated against recombinant HcProRS using a fluorescence-based thermal shift assay. Compound binding usually helps to stabilize the protein due to protein-ligand interactions, resulting in an increase of the melting temperature (T_m_) of the target protein during the thermal denaturation process [23]. Each compound was pre-tested at 100 µM concentration against 3.45 µM HcProRS in the absence and presence of 1 mM Pro. The ∆T_m_ was calculated based on the difference between the T_m_ values of HcProRS with and without compound. In the absence of Pro, only two of the 17 tested compounds, **4h** and **4j**, slightly increased the T_m_ by 0.9 and 2 °C, respectively, while the remaining compounds did not change or slightly decreased the T_m_ (Figure 3 and Table 1). In the presence of the natural substrate Pro, the T_m_ of HcProRS increased by 4 °C. Normalized by this control, the presence of five of the 17 compounds, including **4h** (R = 2-Cl) and **4j** (R = 2-CF_3_), significantly improved the thermal stability of HcProRS resulting with ∆T_m_ values in a range of 2 to 7 °C suggesting these compounds are likely binding to HcProRS in a Pro-dependent manner (Table 1). In a second-tier characterization, the T_m_-based EC_50_ values of these five hits were determined. These compounds demonstrated an activity range varying between lower micromolar (3.77 µM) for the compounds based on scaffold **3** up to 91.11 µM for derivatives of the series **4** compounds (Table 1 and Figure S2). Using the previously reported inhibitor **2a** (100 µM) as the reference, we observed a significant increase of the T_m_ of HcProRS by 7 °C (EC_50_ = 30.35 ± 3.63 µM) and 13.15 °C (EC_50_ = 3.74 ± 0.67 µM) in the absence or presence of Pro, respectively (Table 1 and Figure S2). This is in good agreement with the prior reported improved binding affinity and inhibitory activity of **2a** in the presence of Pro when compared to the corresponding values obtained without Pro [16]. Therefore, taking the limitation of TSA measurements into account, these results suggested that our best compound **3b** and the confirmed inhibitor **2a** behave similarly in binding the enzyme.

Comparison of the full datasets show that the single ring compounds PZA, POA and 3-NH_2_-POA have no effect on T_m_. In 3-benzylamino derivatives of general structure **4**, modification at the ortho-position appeared to be better accepted than at meta- and para-positions. The EC_50_ values of **4h** (R = 2-Cl) and **4j** (R = 2-CF_3_) proved 6-fold better than R = 2-CH_3_ in compound **4d**, indicating that the electron withdrawing properties might be beneficial for binding. Further modification of the C2-carboxamido moiety with a second ortho-methylbenzyl group (**3c**) decreased the EC_50_ 12.4-fold with respect to **4d**, suggesting the modifications of both C3-amino and C2-carboxamido groups of the 3-aminopyrazinamide scaffold are preferable for binding. Similar phenomenon was also observed for the 3.6-fold lower EC_50_ of double substituted compound **3b** (R = 2-Cl) when compared with the single substituted **4h**.

### 2.3. Structural Studies of the Binding Mechanism of the Compounds with HcProRS

**Figure 4:**
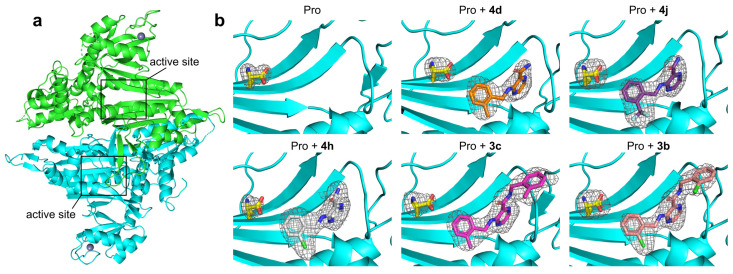
Crystal structures of HcProRS complexes. (**a**) Overview of HcProRS structure in the asymmetric unit. The overall structure is shown as a cartoon representation with chain A and chain B coloured in green and cyan, respectively, while Zn^2+^ ions are shown as grey spheres. (**b**) Omit maps of ligands in the aminoacylation site of chain B of HcProRS. From left to right, the structures are shown in the order of decreasing EC_50_ values. The maps, contoured at 3 σ, were calculated in phenix.polder [24] and shown as grey mesh representations. Pro, compound **4d**, **4j**, **4h**, **3c** and **3b** are shown as sticks and coloured in yellow, orange, purple, white, magenta and salmon, respectively.

To further clarify the binding mechanism of the identified hits, we determined five co-crystal structures of HcProRS in complex with the corresponding compound and Pro at a resolution range of 2.2–2.7 Å (Figure 4 and Table 2). Compared with the reported ligand-free HcProRS structure (PDB ID: 4K86), our structures have two macromolecules, corresponding to the biologically active homodimer, in the asymmetric unit (Figure 4a). In most cases, the calculated electron density map showed a molecule of the respective compound bound in both active sites of the dimeric HcProRS. However, compound **4d** can only be unambiguously built in one chain while showing weak density in the other (Figure 4b and Figure S3). This is consistent with the observed higher EC_50_ for this compound in TSA measurements compared with the other four identified hits (Table 1). These ternary complexes confirmed that these 3-aminopyrazinamide-based compounds share a similar binding pattern with the validated inhibitor **2a**, occupying the ATP binding pockets with Pro found in the amino acid binding site (Figure 4b).

**Figure 5:**
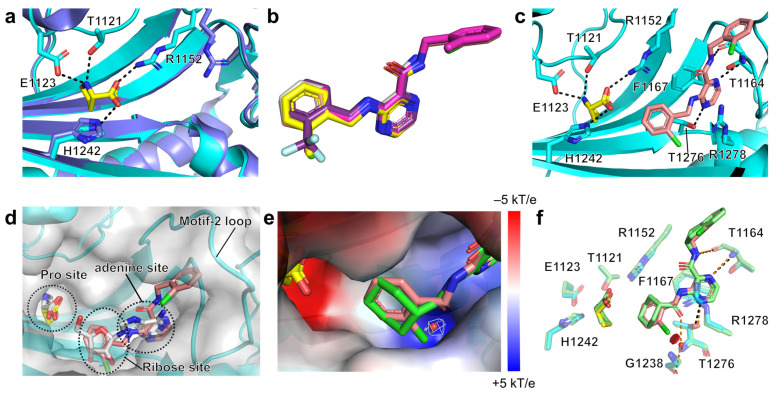
Structural comparison between different ligand-bound structures. (**a**) Superposition of ligand-free (blue; PDB ID: 4K86) and Pro-bound (cyan) HcProRS structures. The important protein residues making directly polar interactions with the substrate Pro are shown as sticks representations. H-bonds and the salt bridge are shown as black dashed lines. (**b**) Protein-based superposition of all five 3-aminopyrazinamide-based ligands. (**c**) The interactions between HcProRS and compound **3b**. The residues interacting with Pro and **3b** are shown as sticks and H-bonds are shown as black dashed lines. (**d**) Superposition of ternary complexes HcProRS:Pro:**3b** (salmon) and HcProRS:Pro:adenosine (grey, PDB ID: 4K87). Pro, adenine and ribose binding sites were highlighted with black dashed circles. The backbone structure of HcProRS:Pro:**3b** is shown as cyan cartoon representation surrounded with a transparent surface. Ligands are shown as sticks. (**e**) Electrostatic surface potential of the ligand binding site of HcProRS:Pro:**3b**. Compound **2a** (green, PDB ID: 5VAD) was superposed onto **3b** (salmon) and the structured water molecule of **2a**-bound structure is shown as a red sphere that is covered with electron density map contoured at 1 σ and shown as white mesh line. (**f**) Superposition of HcProRS:Pro:**3b** (salmon) and HcProRS:Pro:**2a** (green) structures. It shows both compounds bind in the same ATP binding site of HcProRS in a Pro-dependent manner. The H-bonds of former and latter structures are shown as black and orange dashed lines, respectively. The water molecule of **2a**-bound structure is shown as a red sphere. The 2-Cl of the C3-substituted moiety is clearly sterically excluding the binding of the structured water as seen by the calculated potential steric clash (calculated in Pymol and depicted as a red disk).

Superimposition of two chains in the HcProRS complex shows a high degree of similarity as evidenced by the overall mean all-atom RMSD value of 0.373 ± 0.103 Å. Therefore, for the later structural description, we only focus on one monomer, in this case chain B due to the weak electron density of **4d** in chain A (Figure S3). Comparison of Pro-bound and apo-form HcProRS (PDB ID: 4K86) structures shows that the binding of Pro induces the movement of the side chain of Arg1152, a motif-2 invariant residue found in all class II aaRSs [25], away from the ATP binding site to form a salt bridge between the carboxylic group of Pro and guanidino moiety of Arg1152 (Figure 5a). This subsequently provides space to accommodate the binding of pyrazinamide-based compounds in the ATP binding pocket which gives a good rationale why these compounds only show binding in the presence of Pro as seen in the TSA measurements (Table 1).

Superposition of the ligand-bound structures showed that the five compounds adopt a similar conformation (Figure 5b). Detailed analysis of the best compound **3b** highlights that the class II signature motif-2 (residues 1142–1176) and motif-3 (residues 1269–1287) of HcProRS form multiple interactions with this compound. The pyrazine ring is sandwiched by Phe1167 via π-π stacking interaction and Arg1278 via cation-π interaction. The N4 of pyrazine ring makes one direct H-bond with the side chain hydroxyl group of Thr1276 in motif-3. The nitrogen atom N9 of the carboxamide moiety (H-bond donor) and N1 of the pyrazinamide core (H-bond acceptor) establish two H-bonds with the backbone oxygen and nitrogen of Thr1164 in motif-2, respectively (Figure 5c). Moreover, the ortho-chlorobenzyl moiety at C3-amine extends into the ribose binding site of ATP compared with the adenosine-bound HcProRS structure (Figure 5d, PDB ID: 4K87). The ortho-chlorobenzyl moiety attached to the C2-carboxamide is located in a cavity surrounded by residues of the motif-2 loop region and is stabilized via van der Waals interactions (Figure 5d). Despite being non-specific, the interactions of the substituent at C2-carboxamide clearly enhance the binding affinity which can be evidenced by the decreased TSA EC_50_ values of compounds **3b** and **3c** compared to **4h** and **4d**.

As indicated by docking and TSA studies, there is a clear preference for binding of compounds having an ortho-substituted benzyl ring at C3-amine instead of meta- or para-substitution. The binding region of the C3-amine benzyl ring is a β-strand backbone with hydrophobic characteristics. In addition, due to its position at the N-terminus of conserved motif-3 α-helix, the inherent dipole of the α-helix makes the electrostatic potential of this zone of HcProRS overall positive. The positive electrostatic potential is especially profound in a small cavity accepting the ortho-substituent (Figure 5e). Comparisons of inhibitor **2a** (PDB ID: 5VAD) and adenosine-bound (PDB ID: 4K87) structures of HcProRS revealed a structured water molecule is placed in this cavity, bridging the interactions between the ligands (carboxamidic oxygen of **2a** and 2′-OH and 3′-OH of ribose of adenosine) and Thr1276 and Gly1238 (Figure 5e,f). However, in our ligand-bound structures, no electron density of equivalent water is observed, and the ortho-substituents (2-Cl, 2-CF_3_ and 2-CH_3_) of our compounds seem to replace this structured water. Combined, the positive electrostatic potential of this binding pocket indicated that an electronegative substituent at ortho-position of the C3-amine benzyl ring is likely better than neutral or electron-deficient substituents. This is evidenced by the 7-fold better binding potency of 2-Cl and 2-CF_3_ substituted compound **4h** and **4j** compared with 2-CH_3_ substituted **4d** and 2-fold lower EC_50_ of **3b** (2-Cl) relative to **3c** (2-CH_3_) (Table 1). Superposition of compound **3b**-bound structure onto the previously reported inhibitor **2a** (PDB ID: 5VAD) bound HcProRS complex either by aligning the protein structure (RMSD^Cα^ of 0.362 Å over 383 residues) or by superposing the atoms of the pyrazinamide core (RMSD of 0.111 Å), shows that both compounds have very similar conformations. Therefore, **3b** mimics almost all the important interactions of compound **2a** with the exception of the water-mediated H-bonds formed by carbonyl oxygen in the C3-substituted part of **2a** (the carbonyl oxygen atom is missing in our compounds) (Figure 5f). This experimentally determined binding mode of **3b** is also fully consistent with our predictions based on docking (Figure S4).

### 2.4. Molecular Dynamics (MD) Simulations

**Figure 6:**
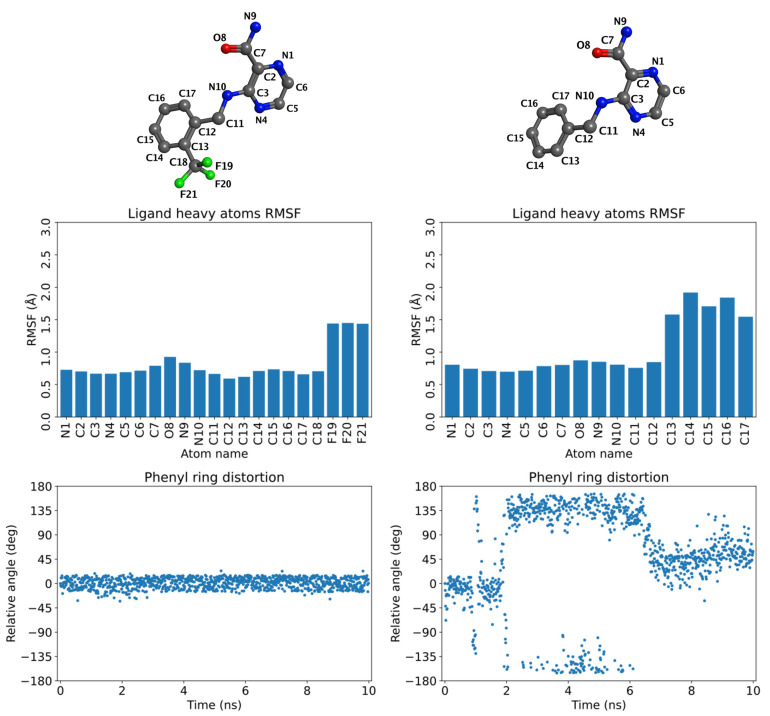
Stability of compounds **4j** (**left**) and **4a** (**right**) in HcProRS by MD simulations.

In an attempt to explain the preference for binding compounds with ortho-substitution on the benzyl ring as suggested by TSA and crystallographic data discussed above, we ran 10 ns MD simulations of the respective ternary complexes. Simulations were performed for 2-substituted compounds **4d** (R = 2-CH_3_), **4h** (R = 2-Cl), **4j** (R = 2-CF_3_), and compared to the results for unsubstituted **4a** (R = H). The initial model for the complex of **4a** was created from the complex of **4h** by simple alchemical transformation of the ligand (replacement of the 2-Cl substituent with a hydrogen). The stability of the ligand’s pose in time was judged by calculating the root mean square fluctuation (RMSF) values for individual heavy atoms of the ligand. Additionally, the stability in terms of the position of the pyrazinamide core was assessed by monitoring the distances of the H-bond forming atoms of the ligand and the receptor, covering all three above-described H-bonds (to carboxamidic hydrogen and to pyrazine N1 and N4). We also observed the conformational rotation of the benzyl plane that was expressed by relative angle to the original state. As shown in Figure 6, the comparison between 2-substituted **4j** (R = 2-CF_3_) and unsubstituted **4a** demonstrated that both ligands were stable in their pyrazinamide part. However, the ortho substituent in **4j** led to the stabilization of the benzyl ring in the original conformation, indicated by small RMSF values for carbon atoms C12-C17 and by negligible changes in the relative conformation angle of the benzyl plane. In contrast, the benzyl ring of the unsubstituted derivative **4a** is predicted to be much more flexible, with the angle oscillating back and forth approximately in the range of −135° to +135°. For full results, see Figures S5 (**4a**) and S6 (**4j**). The similar stabilization of the benzyl ring was also detected for the other studied 2-substituted compounds **4d** and **4h** (Figures S7 and S8).

The results of MD simulations confirm our hypothesis that 2-substitution of the benzyl ring stabilizes the ligand in the ATP-binding site of HcProRS. In the MD-stabilized conformation, which is identical with the crystallographic conformation, the ortho substituent is located in a water binding site as seen in the **2a**-bound structure. Our in silico experiments and the results of the thermal shift assay jointly suggest the ortho-substitution to be favourable. This is further confirmed by the fact that all compounds which we succeeded to co-crystallize with HcProRS bear such an ortho substituent.

### 2.5. Potential Selectivity of Title Compounds towards HcProRS over Other Class II aaRS Members

The 20 standard aaRSs are split into two classes based on two completely different folds of the catalytic cores that originated for the specific recognition and binding of the shared substrate ATP [27,28,29]. The catalytic domain of class I adopts a Rossmann fold while that of class II is organized as a six-stranded β-sheet. Although the binding mode of ATP is strictly conserved in each aaRS class, as ATP-competitive aaRS inhibitors, cladosporin has been reported to specifically target LysRS rather than other class II aaRSs [30] and compound **2a** reportedly also demonstrated 2500-fold selectivity for HcProRS versus another class II member ThrRS. Since our compounds share a similar binding mechanism with compound **2a**, we were encouraged to examine the potential selectivity of our compounds using the available structures of human class II tRNA synthetases.

**Figure 7:**
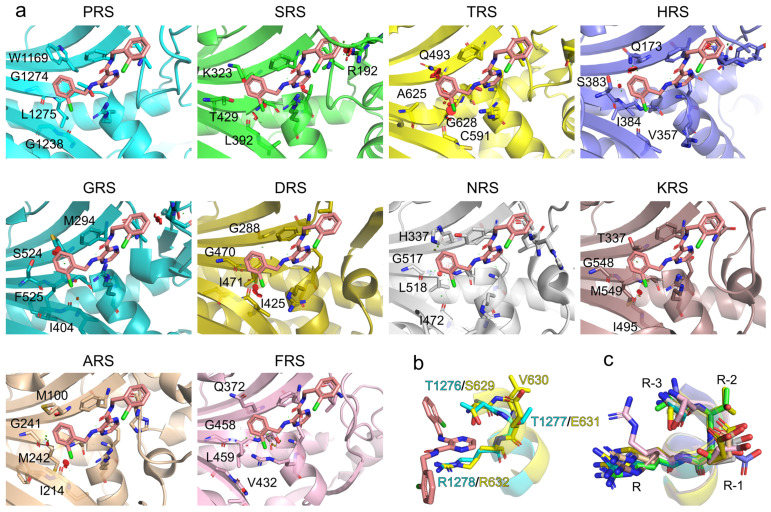
Structural analysis of potential selectivity of HcProRS inhibitors. (**a**) Models of compound **3b** in HcProRS and the other nine human class II aaRSs. Crystal structures of human SerRS (PDB ID: 4L87), ThrRS (PDB ID: 4HWT), HisRS (PDB ID: 4G84), GlyRS (PDB ID: 2ZT5), AspRS (PDB ID: 4J15), AsnRS (PDB ID: 5XIX), LysRS (PDB ID: 6CHD), AlaRS (PDB ID: 4XEM) and PheRS (PDB ID: 3L4G) are used for superposition. All protein structures are shown as cartoon representations with corresponding interacting residues shown as sticks. The red discs represent potential VDW overlaps or steric clashes between **3b** and aaRS residues as calculated in Pymol. (**b**) Superposition of the N-terminus of motif-3 α-helix of HcProRS:Pro:**3b** (cyan) and human ThrRS (yellow; PDB ID: 4HWT). The N-terminus of motif-3 α-helix in HcProRS is one residue shorter than that in ThrRS. (**c**) Superposition of N-terminus of motif-3 α-helix of all other nine human class II aaRSs. Counting from the invariant stacking arginine of motif-3 showed that all human class II aaRSs (with an exception of HcProRS) have four residues at the N-terminus of this α-helix. This structural difference may affect the binding of the pyrazinamide moiety.

Taking compound **3b** as an example, we modelled **3b** in the ATP binding site of all other nine human class II aaRS structures by superimposing the adenine group of available adenine-containing ligand bound structures with HcProRS:Aze-SA (azetidine-2-carboxylic acid (Aze) coupled to 5′-sulfamoyladenosine (SA) is a prolyl-adenylate analogue; PDB ID: 5V58) complex followed by superimposing HcProRS:Pro:**3b** and HcProRS:Aze-SA structures [31]. To thoroughly examine the molecular mechanisms defining the potential family selectivity of **2a** and its derivative **3b**, we split the ligand molecule into three fragments: the C3 substituent, pyrazinamide ring and C2-carboxamide moiety (Figure 2). The former two parts are found in all five confirmed hits and **2a**. In HcProRS, the subpocket binding the 2-substituted benzyl at C3-amine is constituted by Trp1169, Gly1274 and Gly1238. Examination of other class II aaRSs showed these equivalent residues are highly variable. In SerRS, ThrRS, HisRS and GlyRS, belonging to the same subclass (IIa) with ProRS, the corresponding Trp1169 in HcProRS is substituted with polar residues (such as Lys323, Gln493, Gln173 and Met294, respectively) leading to the loss of hydrophobic interaction with the C3-amine benzyl ring of our ligands (Figure 7a). In addition, Gly1274 in motif-3 β-strand is replaced with larger amino acids (Thr429, Ala625, Ser383 and Ser524) that would likely cause a steric clash with the benzyl ring. The Gly1238 is also replaced with residues with side chains (Leu392, Cys591, Val357 and Ile404). Despite these side chains do not make direct contacts with the compound, their presence will immobilize backbone which restricts the carbonyl group pointing to and in parallel with the motif-3 β-strand resulting in potential sterically clash with the ortho-substituent of compound (Figure 7a and Figure S9). Therefore, these residue replacements likely lead to unfavourable binding. For other class II members (AspRS, AsnRS, LysRS, AlaRS and PheRS), two out of three residues are different from those in HcProRS, and the residue equivalent to Gly1238 was replaced by isoleucine or valine. Similarly, both could also result in potential steric repulsion (Figure 7a).

The pyrazinamide moiety sits on the top of the N-terminus of motif-3 α-helix. Structural comparison showed that the stacking residues equivalent to Arg1278 and Phe1167 are highly conserved, and that the residue corresponding to Thr1164 (making backbone interactions) is always present in other class II aaRSs, which may provide a general binding for the pyrazinamide core. However, an obvious distinction was observed in the length of the N-terminus of motif-3 α-helix, where three residues (Thr1276, Thr1277 and Arg1278) are present in HcProRS that are replaced by four residues in all other class II tRNA synthetases (Figure 7b,c). This different structural rearrangement may affect the upstream β-strand and the distinct residues in this region may also provide a steric exclusive binding site for the pyrazinamide ring. The 2-substituted benzyl moiety at C2-carboxamide extends towards the motif-2 loop region and only makes non-specific van der Waals interactions. However, both sequence length and mobility of this loop region are highly diverse. It is difficult to predict how this region will be arranged when these compounds would bind. However, in SerRS, due to the presence of the large side chain of Arg192 sitting in the opposite side of motif-2 loop (equivalent to a glycine in HcProRS (Figure 7a)), a similar steric repulsion is observed for the binding of **3b**, suggesting our confirmed binders are unlikely to bind to SerRS. In summary, we generally note subtle differences in the ATP binding site which appear to be important for designing potential selective ATP competitive inhibitors of aaRSs.

## 3. Discussion

Aminoacyl-tRNA synthetases (aaRSs), catalysing the formation of aminoacyl-tRNA, have been explored as targets for the development of antimicrobials [9,32,33,34,35]. However, HcProRS, located at the C-terminal region of a dual function EPRS, is likewise recognized as a promising target for anti-cancer [12,13,14], anti-fibrosis [11,36] and anti-autoimmune response [37] drug development. Up to date, several HcProRS inhibitors were reported. Halofuginone specifically targets both the amino acid site and 3′-end of tRNA binding sites [10] while compounds **2a** [16] and T-3833261 [36] occupy the ATP binding site of HcProRS. Since the HcProRS inhibitory activity of halofuginone can be reverted by increased concentration of Pro [38] and Pro is accumulated in fibrotic tissues [39], the anti-fibrosis activity of halofuginone is hampered. Therefore, the latter two compounds, as ATP-competitive inhibitors, may overcome this issue. However, since class II aaRSs share a very similar ATP binding mechanism, selective inhibition of HcProRS without affecting other class II members is necessary for drug development.

Compound **2a** demonstrated potent HcProRS inhibitory activity with an IC_50_ value of 12 nM using ATP/PPi exchange assay in vitro and potential selectivity towards HcProRS over the class II ThrRS, without further follow-up studies. Virtual screening of our in-house library of synthetic small molecules using compound **2a**-bound structure as the initial model, identified two general structures of substituted 3-aminopyrazinamide (**3**, **4**) [17,18], which were predicted to bind to HcProRS with the same binding mode as **2a** and with high affinity. Selected compounds were subjected to a thermal shift assay. Five compounds showed clearly increased thermal stability of HcProRS in the presence of Pro, indicating these compounds are Pro-dependent binders of HcProRS similar to compound **2a**. This was further proven by the co-crystal structures of HcProRS in complex with a respective compound and Pro. Pro binding induced the movement of the side chain of Arg1152 out of the ATP binding site, providing additional space to accommodate the binding of pyrazinamide derivatives. The pyrazinamide core mimics the adenine of ATP while the C3-substituent replaces the ribose of ATP via hydrophobic interactions with the active site of HcProRS. The modifications at the C2-carboxamide extending toward the motif-2 loop region of HcProRS improved the binding affinity which can be evidenced by the decreased EC_50_ values for **3b** and **3c** (R = 2-Cl; 2-CH_3_) compared to those of **4h** and **4d** (R = 2-Cl; 2-CH_3_) having unsubstituted carboxamide at C2.

High class selectivity against HcProRS rather than simultaneous targeting of other class II members is fundamental for therapeutic applications of aaRS inhibitors. Despite ATP having a very similar binding pose in class II aaRSs, the subtle differences between active sites in different class II aaRS enzymes may contribute to designable selectivity. Modelling compound **3b** in all other class II aaRS structures pointed out that three residues surrounding the ortho-substituted benzyl moiety at C3 of the pyrazinamide core and making hydrophobic interactions with the ligand, were replaced with either polar or larger residues in other class II enzymes. Therefore, these latter enzymes cannot form stable interactions with the C3-substituted moiety. In addition, the specific arrangement of the N-terminus of motif-3 α-helix in HcProRS (being one residue shorter than that in other class II members) is responsible for the exact positioning of the pyrazinamide ring, which may also contribute to its selectivity.

In conclusion, a combination of virtual screening with biophysical and crystallographic studies successfully identified five new pyrazinamide-based HcProRS ligands that specifically compete with the binding of the ATP substrate in a Pro-dependent manner. This research not only applies to the potential repurposing of old compounds for new applications, but also provides the possibility to further improve the binding affinity and potential selectivity of HcProRS inhibitors by structure-based drug design particularly at the C3 substituent. This should be followed by inhibitory activity measurements and whole cell assays. Taken together, our study leads to the successful exploration and broadening of the chemical space around the confirmed HcProRS inhibitor reported by Adachi. These may provide new drug candidates for the clinical treatment of ProRS-associated diseases.

## 4. Materials and Methods

### 4.1. Protein Preparation

The DNA sequence encoding human ProRS (HcProRS) encompassing residues 1001–1512 (UniProt accession ID: P07814) was amplified by PCR from a cDNA library produced from HEK293T cells. The amplified gene was cloned into the in-house pETRUK vector, a derivative of pETHSUL [40] that yields a fusion protein with a SUMO tag at the N-termini for expression in *E. coli* Rosetta 2 (DE3) pLysS cells. After culture by using auto-induction media [41], cells were harvested and lysed by sonication in cation exchange buffer A containing 25 mM Hepes pH 8, 200 mM NaCl and 5 mM β-mercaptoethanol supplemented with 1 mM MgCl_2_ and 100 U Cryonase (Takara). The lysis was cleared by centrifugation at 18,000× *g* for 30 min and the resulting supernatant was applied to a 5 mL Hitrap SP column (Cytiva, Marlborough, MA, USA). The fractions corresponding to the SUMO-fused HcProRS were collected followed by SUMO hydrolase treatment to remove the SUMO tag. This combined mixture was dialyzed in buffer containing 10 mM Tris pH 7, glycerol 10% (*w*/*v*) overnight to remove the salt which was then loaded onto a 5 mL Hitrap SP column (Cytiva, Marlborough, MA, USA) to remove the SUMO tag and SUMO hydrolase. The flow through containing HcProRS was further purified by anion exchange chromatography and gel filtration. Purified HcProRS was concentrated to 45 mg/mL in the final buffer (10 mM Tris pH 7, 100 mM NaCl and 2.5 mM β-mercaptoethanol) and stored at −80 °C.

### 4.2. Crystallization of HcProRS Complexes

For crystallization of HcProRS complexes, the purified protein at 30 mg/mL in 10 mM Tris pH 7, 100 mM NaCl, 2.5 mM β-mercaptoethanol was incubated with 10 mM L-proline and 12% (*v*/*v*) DMSO in the absence or presence of 2 mM compound on ice for 1 h before setting up crystallization using the Microbatch method. Crystals were grown in a Terasaki Microbatch plate by mixing 1 µL the pre-mix with 1 µL of reservoir solution containing 0.25–0.4 M SrCl_2_, 15–20% (*w*/*v*) PEG3350 and 100 mM HEPES pH 7.5. The drops were covered with paraffin oil. Suitable crystals were mounted and flash frozen in liquid nitrogen directly from the plate.

### 4.3. Data Collection and Structure Determination

All X-ray diffraction datasets were collected from cryo-cooled crystals at Synchrotron radiation facility and processed by using autoPROC software package [42]. The initial model of structure was determined by molecular replacement with reported HcProRS:Pro:**2a** structure (PDB ID: 5VAD) using the program PHASER from the Phenix program suite [26]. Subsequent structure refinement was conducted in Phenix.refine and the model was manually built in COOT [43]. Data collection and refinement statistics are summarized in Table 2, and all structural figures were generated using PyMol (http://www.pymol.org, version 2.0.4). All the crystal structures were deposited in PDB and the related accession codes are as follows: 7OSY, 7OSZ, 7OT0, 7OT1, 7OT2 and 7OT3.

### 4.4. Thermal Shift Assay

A total of 0.2 mg/mL HcProRS (corresponding to 3.45 µM) was incubated with various concentrations of different compounds in the absence or presence of 1 mM L-Pro with 1x thermal shift dye (Life Technologies, California, USA) in reaction buffer containing 50 mM HEPES pH 7.0, 150 mM KCl and 10% (*v*/*v*) ethylene glycol. Aliquots (20 µL) were transferred to a 96-well PCR plate in triplicate. After centrifugation to remove air bubbles, the plate was measured in Applied Biosystems^®^ Protein Thermal Shift machine and subjected to a thermal gradient from 4 °C to 95 °C with the increasing rate of 0.05 °C/s. The fluorescence was detected by Protein Thermal Shift™ Software (Life Technologies, California, USA). Data analysis was performed by Boltzmann sigmoidal fitting to calculate melting temperature (T_m_).

### 4.5. In Silico Studies

Inputs for molecular docking and MD were prepared in Molecular Operating Environment (MOE), 2020.09 (Chemical Computing Group ULC, Montreal, QC, Canada). The docking was run in MOE. MD simulations were run in NAMD2 [44] (version GIT20190909) and analysed in VMD version (1.9.4a51) [45]. For experimental details, see the Supplementary Material.

## Figures and Tables

**Table 1:** The docking scores, thermal stabilization and EC_50_ values of studied compounds.

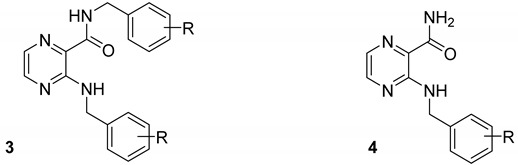									
**Code**	**Structure/R**	**Without L-Pro**		**With 1 mM L-Pro**			**Docking ^5^**		
		**Tm ^1^** **(°C)**	**∆Tm ^2^** **(°C)**	**Tm ^3^** **(°C)**	**∆ Tm ^4^** **(°C)**	**EC_50_** **(µM)**	**S**	**RMSD** **(Å)**	**Pose**
HcProRS	/	56.15	/	60.20	/	/	/	/	/
POA	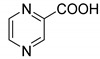	55.52	−0.63	59.06	−1.14	ND	ND	ND	ND
PZA	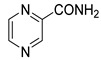	56.06	−0.09	59.04	−1.16	ND	ND	ND	ND
3-NH_2_-POA	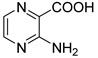	55.87	−0.28	59.50	−0.70	ND	ND	ND	ND
**3a**	4-F	55.25	−0.90	58.21	−1.99	ND	−8.672	0.540	1
**3b**	2-Cl	56.05	−0.10	64.92	+4.72	3.77 ± 0.85	−9.240	0.425	1
**3c**	2-CH_3_	55.25	−0.90	62.72	+2.52	7.34 ± 1.66	−9.283	0.443	1
**4a**	H	55.80	−0.35	59.36	−0.84	ND	−6.603	0.308	1
**4b**	3-Cl	55.38	−0.77	60.42	+0.22	ND	−6.666	0.964	2
**4c**	3,4-diCl	54.57	−1.58	57.47	−2.73	ND	−6.959	2.765	no
**4d**	2-CH_3_	55.91	−0.24	63.33	+3.13	91.11 ± 18.39	−6.949	0.247	2
**4e**	4-Cl	55.06	−1.09	58.70	−1.5	ND	−6.824	2.507	no
**4f**	4-OCH_3_	56.34	+0.19	58.33	−1.87	ND	−6.990	2.615	no
**4g**	4-CH_3_	55.76	−0.39	59.09	−1.11	ND	−7.052	2.477	no
**4h**	2-Cl	57.04	+0.89	62.47	+2.27	13.63 ± 3.27	−6.651	0.926	2
**4i**	2-F	55.46	−0.69	60.30	+0.10	ND	−6.777	0.299	1
**4j**	2-CF_3_	58.13	+1.98	67.12	+6.92	15.28 ± 1.04	−7.133	0.706	1
**4k**	2,4-(OCH_3_)_2_	56.08	−0.07	58.48	−1.72	ND	−7.160	0.438	2
**2a** ^6^		63.15	+7.00	73.35	+13.15	3.74 ± 0.67	−9.767	0.190	1

^1^ T_m_ values of HcProRS measured in the absence (see top row, reference value) or presence of compound. ^2^ ∆T_m_ was calculated based on the difference of T_m_ values of HcProRS measured in the presence and absence of compound. ^3^ T_m_ values of HcProRS measured in the presence of compound and 1 mM Pro. ^4^ ∆T_m_ was calculated based on the difference of T_m_ values of HcProRS measured in the presence of both compound and 1 mM Pro and only in the presence of 1 mM Pro. ^5^ S—docking score; RMSD—to the crystallographic pose of the confirmed inhibitor **2a**, calculated for the shared subset of atoms (bolded in Figure 2); Pose—number of the pose (ordered by S) which took the binding mode of the confirmed inhibitor **2a**. ^6^ Compound **2a** reported by Adachi was resynthesized and used as the positive control during TSA measurements.

**Table 2:** Data collection and refinement statistics for the structures of HcProRS complexes.

PDB Code	HcProRS:Pro	HcProRS:Pro:4d	HcProRS:Pro:4h	HcProRS:Pro:4j	HcProRS:Pro:3b	HcProRS:Pro:3c
	7OSY	7OSZ	7OT0	7OT2	7OT3	7OT1
**Data collection**						
Resolution range (Å)	39.3–2.23 (2.31–2.23)	65.41–2.46 (2.548–2.46)	63.09–2.32 (2.403–2.32)	54.91–2.48 (2.569–2.48)	52.36–2.53 (2.62–2.53)	62.51–2.711 (2.808–2.711)
Space group	P 1 21 1	P 1 21 1	P 1 21 1	P 1 21 1	P 1 21 1	P 1 21 1
Unit cell	69.9 88.4 83.8 90 110.2 90	69.7 86.7 83.1 90 110.1 90	71.2 93.5 87.5 90 108.6 90	72.0 92.8 87.9 90 109.0 90	71.9 92.4 87.3 90 108.9 90	70.3 89.0 84.8 90 110.3 90
Unique reflections	47,843 (4596)	33,698 (3333)	45,788 (4523)	38,563 (3849)	35,859 (3530)	26,094 (2642)
Multiplicity	4.8 (4.9)	3.7 (3.8)	3.8 (3.8)	6.7 (7.2)	6.9 (7.0)	3.7 (3.9)
Completeness (%)	98.05 (99.07)	99.48 (99.52)	96.97 (96.14)	99.01 (99.35)	99.05 (98.85)	97.69 (99.36)
Mean *I/σ* (*I*)	11.67 (1.99)	7.03 (1.46)	8.50 (1.52)	14.44 (2.02)	18.10 (1.97)	8.26 (2.24)
Wilson B factor (Å^2^)	46.34	50.69	55.22	67.28	80.11	49.02
*R_merge_*	0.07098 (0.8346)	0.1066 (0.9065)	0.08095 (1.054)	0.06697 (1.023)	0.04557 (0.8425)	0.1284 (0.83)
*R_meas_*	0.07969 (0.9342)	0.1253 (1.055)	0.09489 (1.232)	0.0727 (1.103)	0.04929 (0.9104)	0.1504 (0.9602)
*R_pim_*	0.03575 (0.415)	0.06494 (0.5341)	0.04872 (0.6289)	0.02793 (0.4093)	0.01858 (0.3414)	0.07727 (0.4768)
CC_1/2_	0.999 (0.713)	0.996 (0.711)	0.996 (0.696)	0.999 (0.84)	0.999 (0.781)	0.992 (0.702)
**Refinement**						
Reflections used for R_free_	1940 (209)	1724 (183)	2190 (196)	1865 (186)	1768 (173)	1230 (130)
*R_work_*	0.2090 (0.2637)	0.2200 (0.3128)	0.1966 (0.3146)	0.1936 (0.2928)	0.2180 (0.3048)	0.2140 (0.2871)
*R_free_*	0.2601 (0.3210)	0.2777 (0.3869)	0.2503 (0.3632)	0.2548 (0.3860)	0.2757 (0.3872)	0.2833 (0.3630)
Number of non-H atoms	7653	7646	7847	7764	7581	7631
Macromolecules	7502	7505	7691	7655	7481	7486
Ligands	16(Pro)	16(Pro)/36(**4d**)	16(Pro)/36(**4h**)	16(Pro)/42(**4j**)	16(Pro)/52(**3b**)	16(Pro)/52(**3c**)
Solvent	127	80	100	47	26	69
RMS bonds (Å)	0.003	0.003	0.005	0.006	0.003	0.003
RMS angles (°)	0.91	0.91	0.98	1.09	0.92	0.90
Ramachandran favored (%)	97.28	97.08	98.06	97.34	97.21	96.33
Ramachandran allowed (%)	2.62	2.61	1.94	2.66	2.68	3.67
Average B-factor (Å^2^)	63.57	62.84	71.61	85.06	115.2	52.18
Protein	63.8	62.82	71.84	85.24	115.32	52.26
Ligands	41.78(Pro)	46.88(Pro)/86.19(**4d**)	45.85(Pro)/66.03(**4h**)	57.29(Pro)/68.15(**4j**)	78.54(Pro)/99.88(**3b**)	36.49(Pro)/52.9(**3c**)
Solvent	52.88	53.62	58.58	75.61	113.26	41.84

Statistics were generated using Phenix [26], values in parenthesis correspond to the highest resolution shell.

## Data Availability

The structural datasets generated during the current study are available in the PDB repository (https://www.rcsb.org/) under accession codes: 7OSY, 7OSZ, 7OT0, 7OT1, 7OT2, 7OT3.

## Supplementary Materials

Detailed results and additional description of methods are available online at https://www.mdpi.com/article/10.3390/ijms22157793/s1.
