# Supplementary material for: Towards Novel 3-Aminopyrazinamide-Based Prolyl-tRNA Synthetase Inhibitors: In Silico Modelling, Thermal Shift Assay and Structural Studies
Source: Int J Mol Sci. 2021 Jul 21;22(15):7793. doi: 10.3390/ijms22157793 (PMC8346053; doi:10.3390/ijms22157793)

Towards Novel 3-Aminopyrazinamide-Based Prolyl-tRNA Synthetase Inhibitors: In Silico Modelling, Thermal Shift Assay and Structural Studies

Luping Pang, Stephen D. Weeks, Martin Juhás, Sergei V. Strelkov, Jan Zitko and Arthur Van Aerschot

SUPPLEMENTARY MATERIALS

Contents

Virtual screening by molecular docking 2
Experimental procedure 2
Results 3
Thermal shift assay 5
Crystallographic studies 6
MD simulations assessment of compound stability in HcProRS 7
Experimental procedure 7
Results 8
Structural basis for the potential selectivity of confirmed HcProRS binder 12
Synthesis of 3-(cyclohexanecarboxamido)-*N*-(2,3-dihydro-1*H*-inden-2-yl)pyrazine-2-carboxamide (**2a**, Adachi ligand) 13
NMR spectra of **2a** 14
MS spectra of **2a** 15

Virtual screening by molecular docking

Experimental procedure

In silico calculations were performed in Molecular Operating Environment (MOE), 2020.09 (Chemical Computing Group ULC, Montreal, QC, Canada) under AMBER10:EHT force field.

The ligands for docking were generated from SMILES, using the dominant protomer at pH 7. For two compounds, the abundance of the dominant protomer was 87%, for the rest of the compounds the abundance of the dominant protomer was >99%. 3D coordinates were minimized to RMS gradient 0.01 $kcal.mol^{-1}.Å^{-1}$.

3D structure for the human prolyl-tRNA synthetase (HcProRS) was downloaded from the PDB database (PDB ID: 5VAD). This structure is HcProRS co-crystallized with a pyrazinamide-based inhibitor **2a** and L-proline substrate. The receptor (chain A) was prepared by MOE QuickPrep functionality with default settings. This included correction of structural errors, the addition of hydrogens, calculation of partial charges, 3D optimization of H-bond network (Protonate3D), deletion of water molecules further than 4.5 Å from any receptor or ligand atom, and restrained energy minimization (to RMS gradient of 0.01 $kcal.mol^{-1}.Å^{-1}$) of ligand and pocket residues within 8 Å from the ligand. Subsequently, all solvent molecules were removed, and the L-Pro substrate was defined as a part of the receptor. The binding site was defined based on the original co-crystallized inhibitor and was defined as a set of residues having at least one atom within 4.5 Å from the co-crystalized ligand. Parameters of the MOE docking protocol: Docking stage – Placement method: Triangle Matcher; Score: London dG; retain 30 poses. Refinement stage – Rigid receptor; Score: GBVI/WSA dG; retain 5 poses. Ligand conformations – Rotate bonds.
Using the protocol described above, the original ligand **2a** was successfully redocked (S = -9.767) with RMSD = 0.526 in comparison to the crystallographic pose, the only significant difference being in the flexible cyclohexane ring.

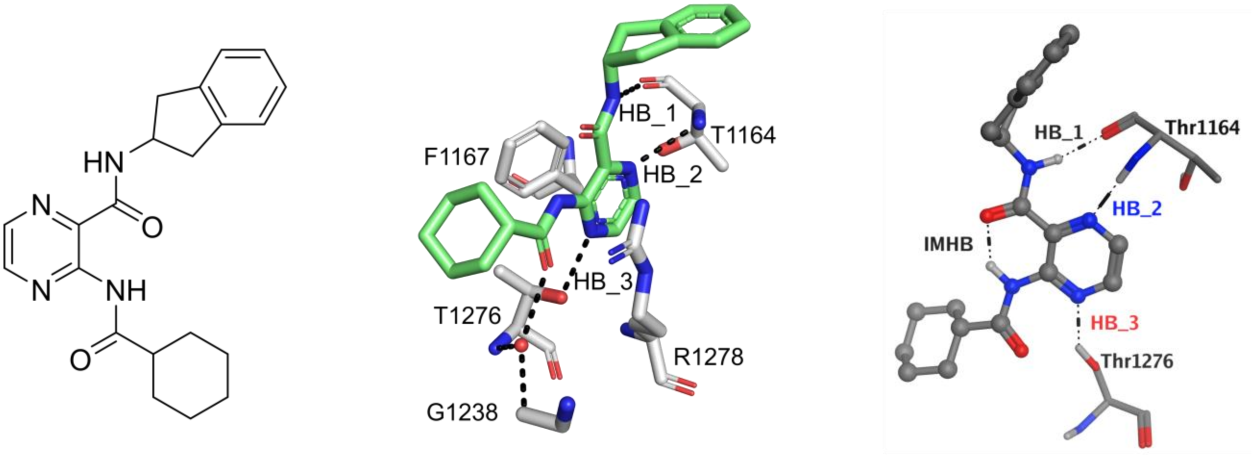

2a | **Crystallographic pose** | **Docking pose**

Figure S1. Annotation of hydrogen bonds as used in Table S1, exemplified on compound **2a**.

Results

Table S1. Results of molecular docking of in-house compounds of general structure **3** and **4**, ordered by docking score, in comparison with confirmed Adachi inhibitor **2a** [a]

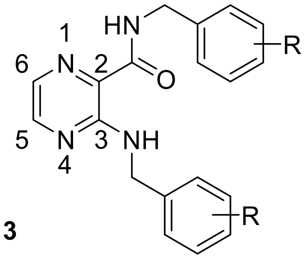

3

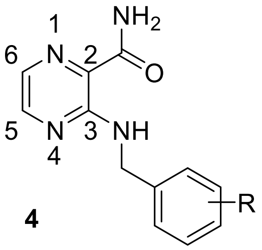

4

Lab code	Article code	R	S	RMSD[b] (Å)	HB_1[c]	HB_2[c]	HB_3[c]	IMHB[c]	Binding mode[d]	Pose No.[e]
LS51	3c	2-CH_3	-9.283	0.443	1	1	1	1	normal	1
LS50	3b	2-Cl	-9.240	0.425	1	1	1	1	normal	1
LS47	3-	2-F	-8.905	0.596	1	1	1	1	normal	1
LS49	3-	3-Cl	-8.863	0.626	1	1	1	1	normal	1
LS46	3-	4-CF_3	-8.805	0.864	0	1	1	0	normal	1
LS48	3-	3,4-diCl	-8.772	0.748	0	1	1	0	normal	1
LS45	3a	4-F	-8.672	0.540	1	1	1	1	normal	1
LS41	3-	4-OCH_3	-8.554	0.849	0	1	1	1	normal	1
LS52	3-	4-CH_3	-8.465	0.685	0	1	1	0	normal	1
LS42	3-	4-Cl	-8.254	0.691	0	1	1	1	normal	1
OJ54	4-	3-NO_2	-7.289	2.709	0	1	1	1	upside down	1
MD680	4-	3-CF_3	-7.224	0.698	1	1	1	0	normal	1
OJ53	4k	2,4-diOCH_3	-7.169	5.890	NA	NA	NA	0	diff	1
OJ52	4j	2-CF_3	-7.133	0.706	1	1	1	1	normal	1
OJ47	4g	4-CH_3	-7.052	2.477	1	1	1	0	upside down	1
OJ46	4f	4-OCH_3	-6.990	2.615	0	1	1	0	upside down	1
MD679	4c	3,4-diCl	-6.959	2.765	0	1	1	0	upside down	1
MD682	4d	2-CH_3	-6.949	0.247	1	1	1	1	normal	2
MD681	4e	4-Cl	-6.824	2.507	1	1	1	0	upside down	1
OJ51	4-	4-CF_3	-6.798	2.880	0	1	1	0	upside down	1
MD678	4b	3-Cl	-6.783	2.817	0	1	1	1	upside down	1
OJ50	4i	2-F	-6.777	0.299	1	1	1	1	normal	1
OJ49	4h	2-Cl	-6.651	0.926	1	1	1	1	normal	2
OJ48	4-	4-NH_2	-6.608	3.698	NA	NA	NA	1	diff	1
MD677	4a	H	-6.603	0.308	1	1	1	1	normal	1
Adachi	2a	NA	-9.767	0.190	1	1	1	1	normal	1

[a] Background colour: yellow – compounds active in the thermal shift assay and successfully co-crystallized; grey – not active in the thermal shift assay; white - tested only in silico (not selected for further studies). NA – not applicable. Red colour indicates deviation from the reference binding mode of the Adachi inhibitor (RMSD >1, absent H-bond, and/or different binding mode).

[b] RMSD – relative to the crystallographic pose of the original inhibitor **2a**, calculated for the shared subset of atoms (bolded in Figure 2 in the main article).

[c] For annotation of the H-bonds, see Figure S1 above. 1 – H-bond is present; 0 – H-bond not present. IMHB – intramolecular H-bond.

[d] Binding mode (relative to the confirmed Adachi inhibitor): normal – equal; upside down – the orientations of C2-

carboxamide and C3-amine of the ligand in the protein structure are inverted; diff – different.
[e] Pose No. – pose ranking by docking score.

Thermal shift assay

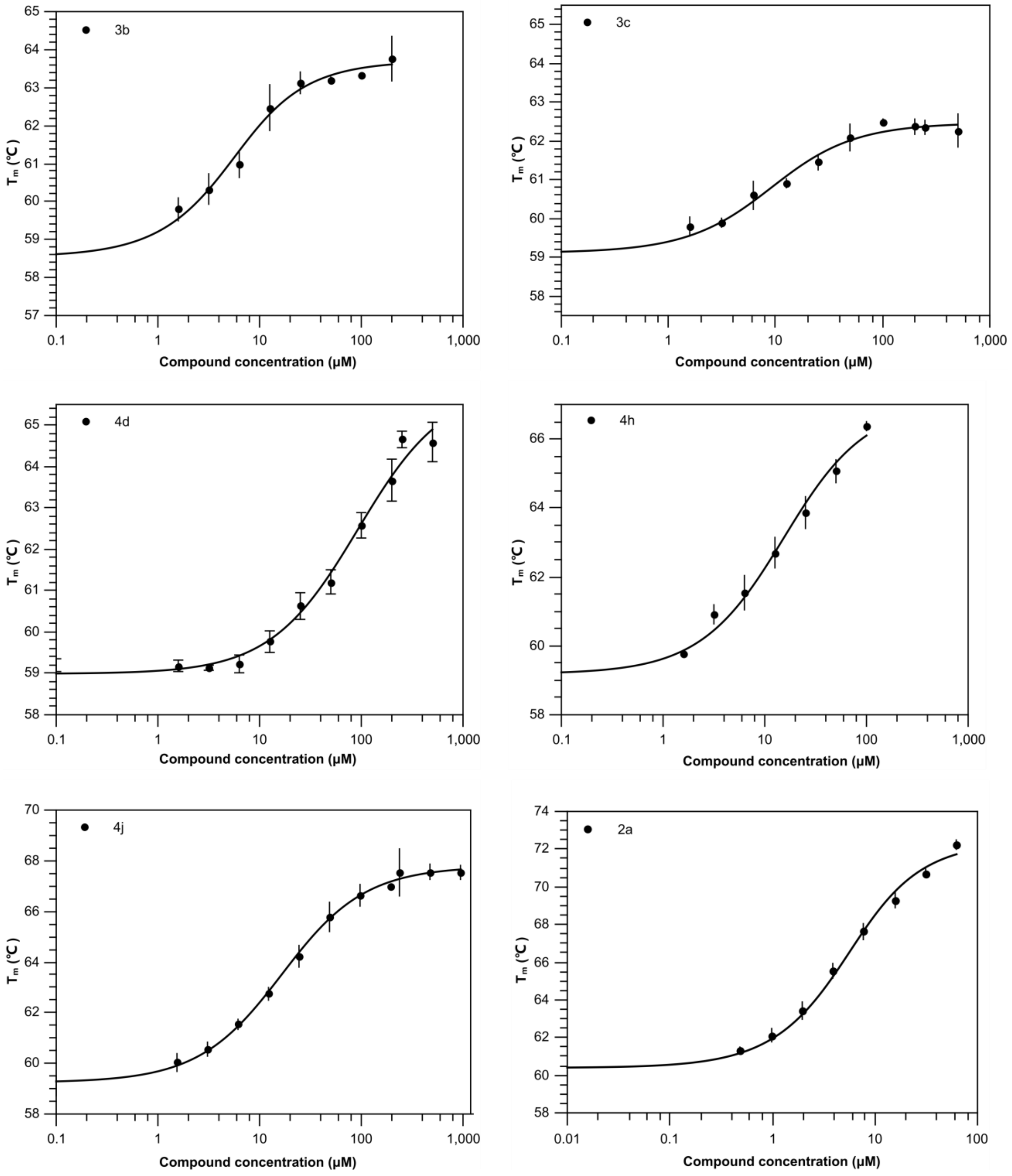

Figure S2. Dose-response melting curves of HcProRS in the presence of compound and 1 mM Pro. The melting temperature (T_m) at each compound concentration was determined by TSA with 3.45 µM of HcProRS. The reported EC_{50} values are based on the fitted curves. All reactions were performed in triplicate, the average values and standard errors are shown.

Crystallographic studies

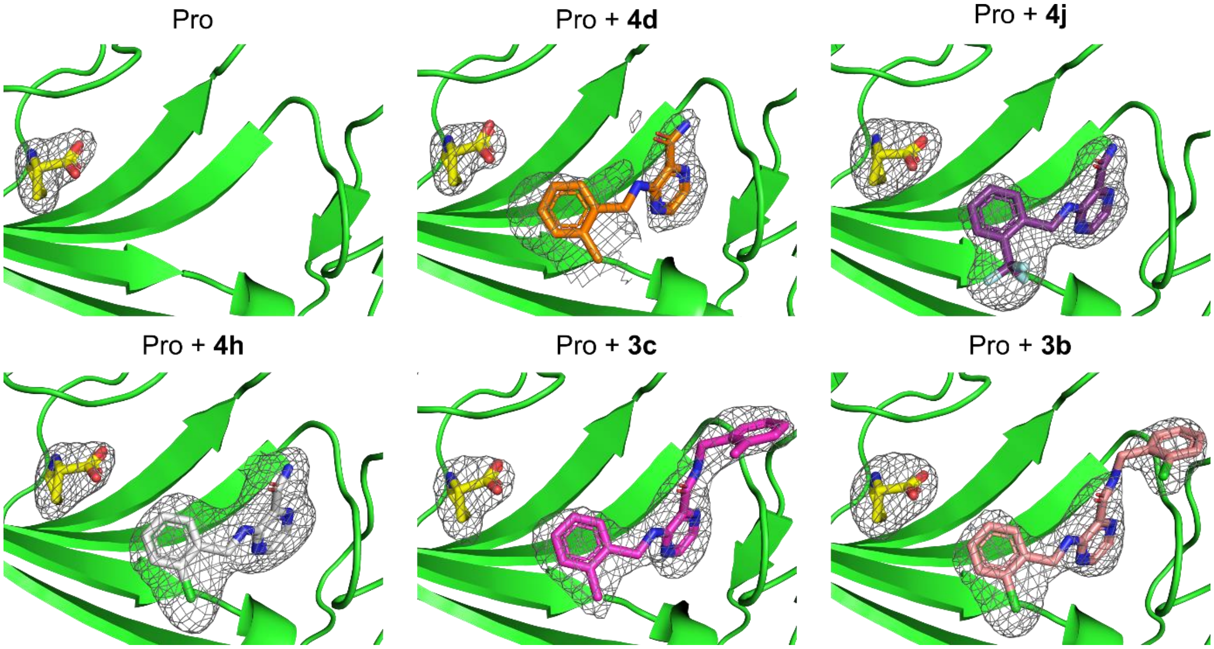

Figure S3. Omit maps of ligands in the aminoacylation site of chain A of HcProRS. From left to right, the structures are shown in the order of decreasing EC_{50} values. The maps, contoured at 3 σ, were calculated in phenix.polder and shown as grey mesh representations. Pro, compound **4d**, **4j**, **4h**, **3c** and **3b** are shown as sticks and coloured in yellow, orange, purple, white, magenta and salmon, respectively.

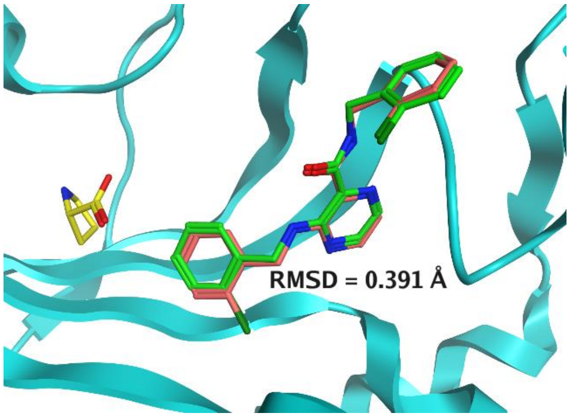

Figure S4. Overlay of the docking pose (green carbons) and crystallographically determined pose (salmon carbons) of **3b**

MD simulations assessment of compound stability in HcProRS

Experimental procedure

Inputs for the molecular dynamics (MD) simulations were prepared in MOE 2020.09 using MOE Protein Preparation utilities. The systems for compounds **4d**, **4h**, and **4j** were prepared from our crystallographic structures of the respective ternary complexes HcProRS:Pro:compound (see the main article for PDB accession codes). The initial model for the complex of **4a** (R = H) was created from the complex of **4h** (R = 2-Cl) by a simple alchemical transformation of the ligand (replace 2-Cl substituent with a hydrogen atom).

Missing or incorrect side chains were added/corrected, and the missing loops were built (none of these issues was in the vicinity of the binding site). Hydrogens were added, the termini were capped, and the system was charged using Amber14:EHT force field. Subsequently, the whole system was treated with Protonate3D utility as implemented in MOE, optimizing the H-bond network as well as assigning appropriate protonation states, side-chain flips etc. for both protein residues and ligands. The system was solvated in TIP3P water box with a 10 Å margin, neutralized, and buffered with NaCl (c = 0.1 M).

MD simulations were run using NAMD2 engine (version GIT20190909) on HPC clusters provided by CERIT-SC and MetaCentrum (Czech Republic). Parameters calculated by Amber14:EHT in MOE were used. As defined in the Amber force field, the 1-4 electrostatics were scaled by a factor of 0.833333. During the simulation, all heavy atom-hydrogen bonds were constrained. SETTLE algorithm was used for water molecules, and other applicable bonds were treated with SHAKE algorithm. Periodic boundaries conditions were used, long-range electrostatics were treated with Particle Mesh Ewald (PME). The cut-off distance was set to 10 Å. Every 10th step was sampled to the final trajectory with a 2 fs timestep. The trajectories were gathered and analysed in VMD 1.9.4a51 and plotted using a Python script.

MD Protocol:

1. **Restrained minimization** - 10 ps - with 1 Å heavy atoms tether. The applied tether was defined as an applied restraint force constant allowing 1 Å radial standard deviation from the reference position.
2. **Unrestrained minimization** - 10 ps
3. **Heating** - 200 ps – gradual heating (from T=10 to T=300 K, 2 Å tether)
4. **NPT Equilibration** - 500 ps (T=300 K, P=1 bar, 2 Å tether)
5. **Production phase** - 10 ns at NPT conditions (T=300 K, P=1 bar).

The temperature was controlled by Langevin dynamics, and the pressure was treated using Nosé-Hoover Langevin piston pressure control, both implemented in NAMD2.

Results

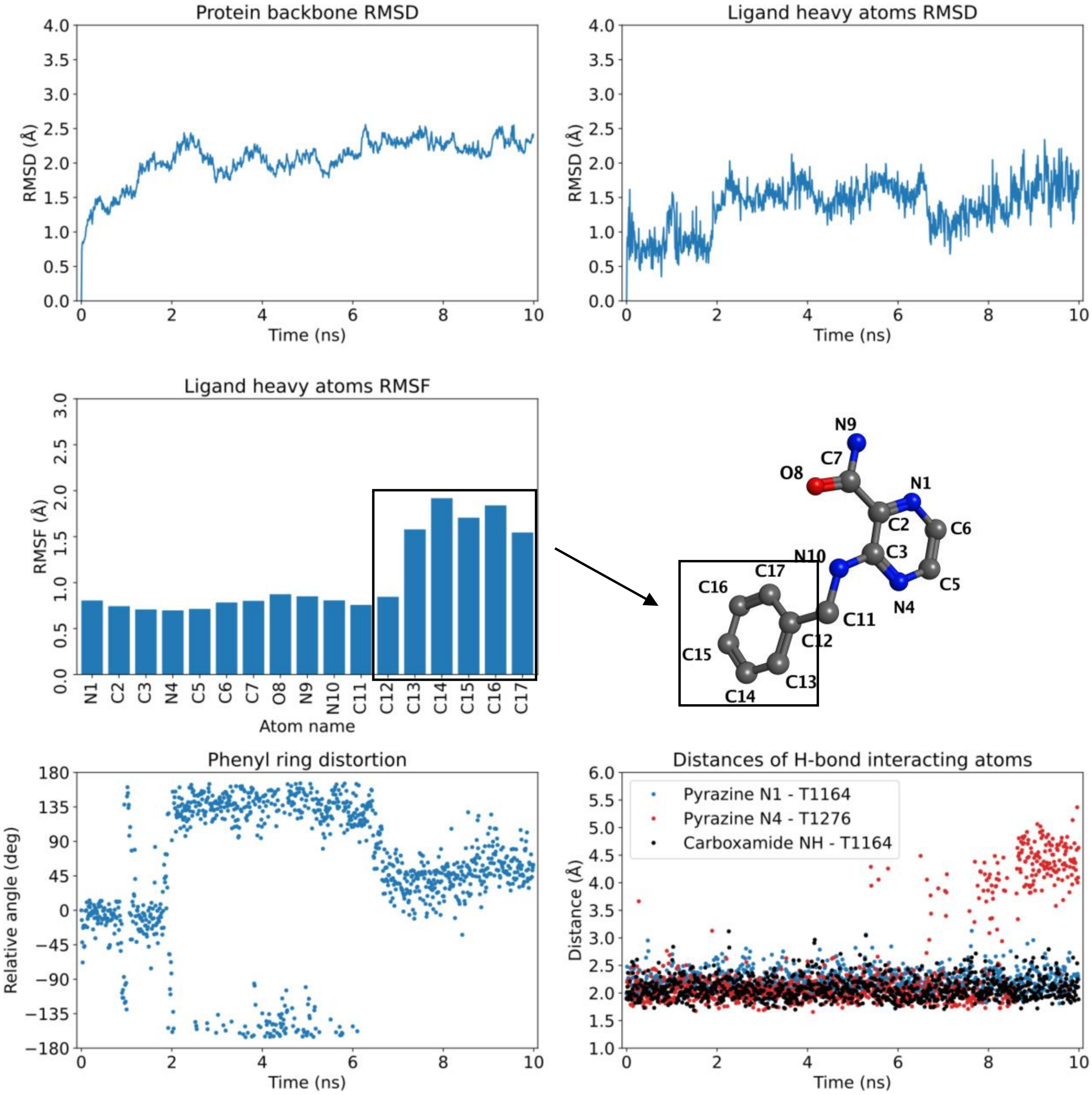

Figure S5. Stability of compound **4a** in HcProRS by MD simulation

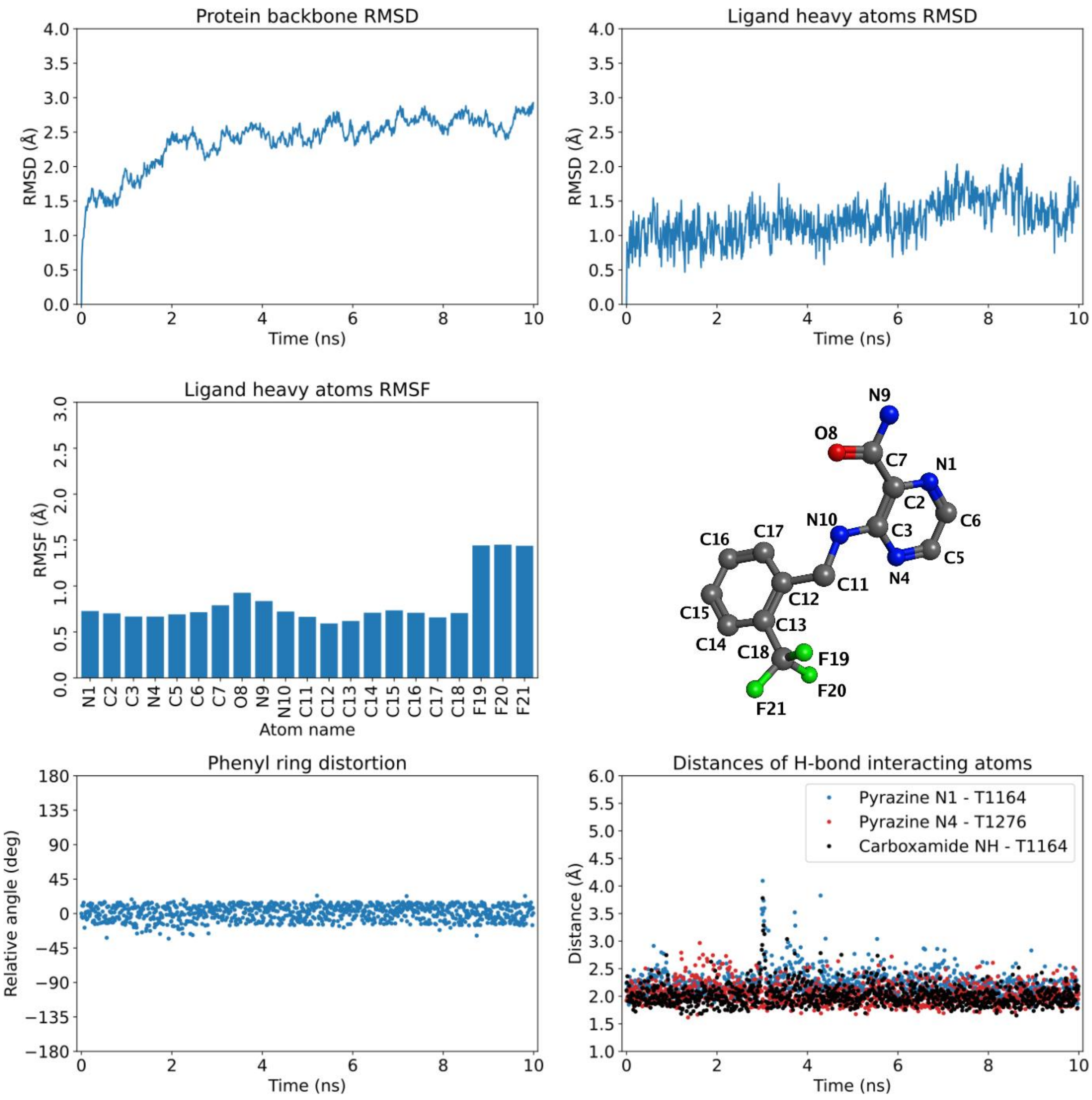

Figure S6. Stability of compound **4j** in HcProRS by MD simulation

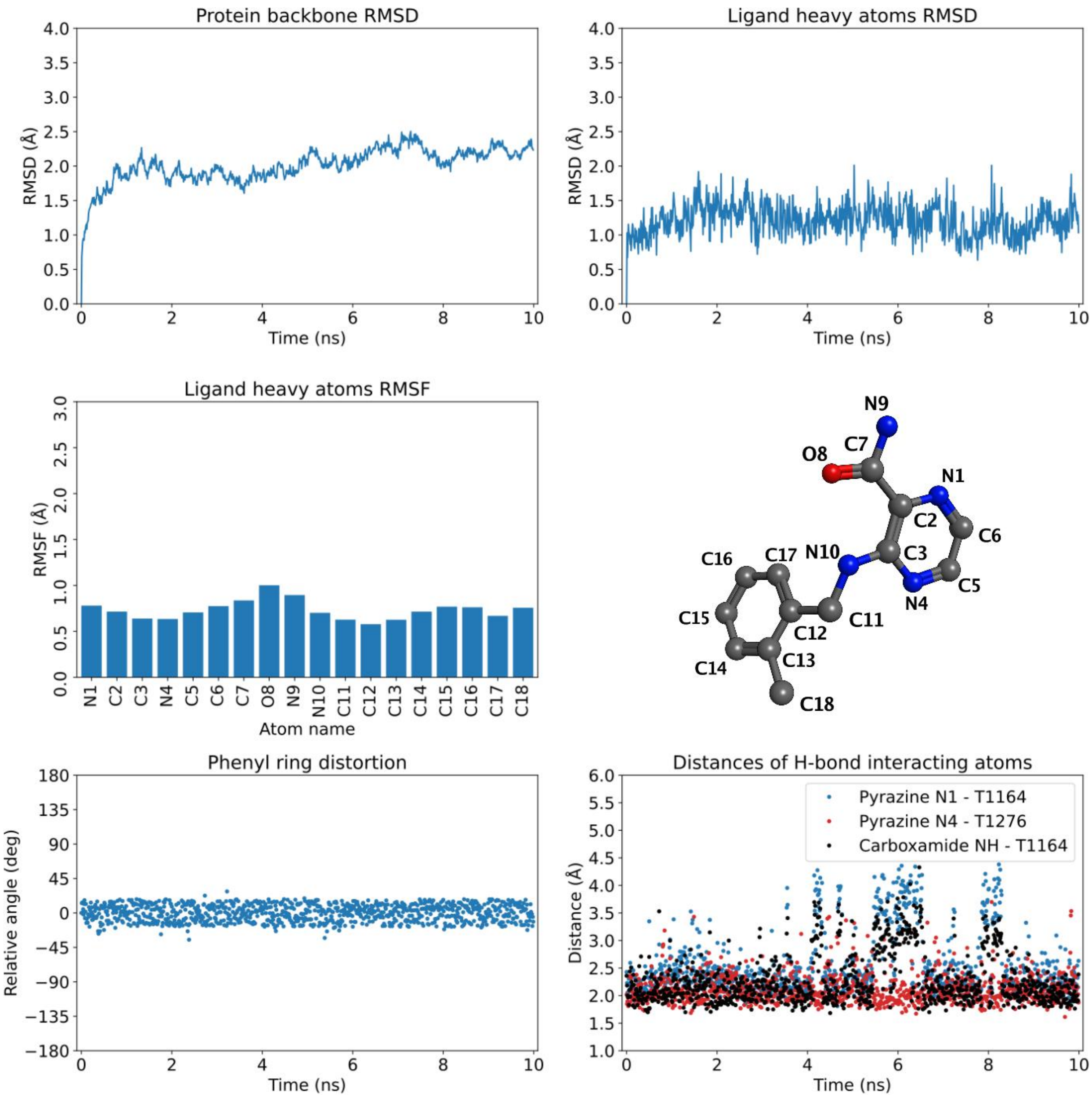

Figure S7. Stability of compound **4d** in HcProRS by MD simulation

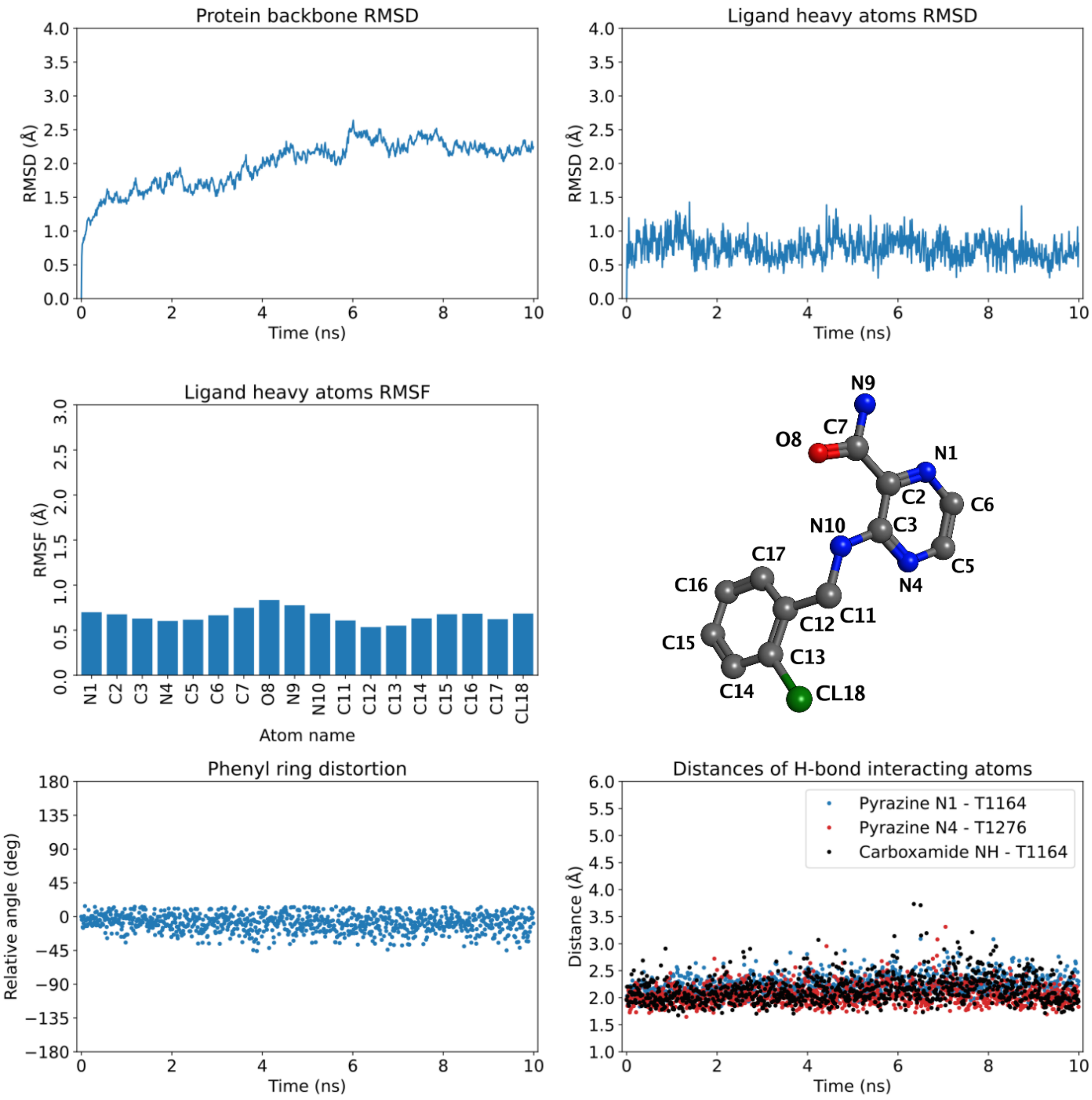

Figure S8. Stability of compound **4h** in HcProRS by MD simulation

Structural basis for the potential selectivity of confirmed HcProRS binder

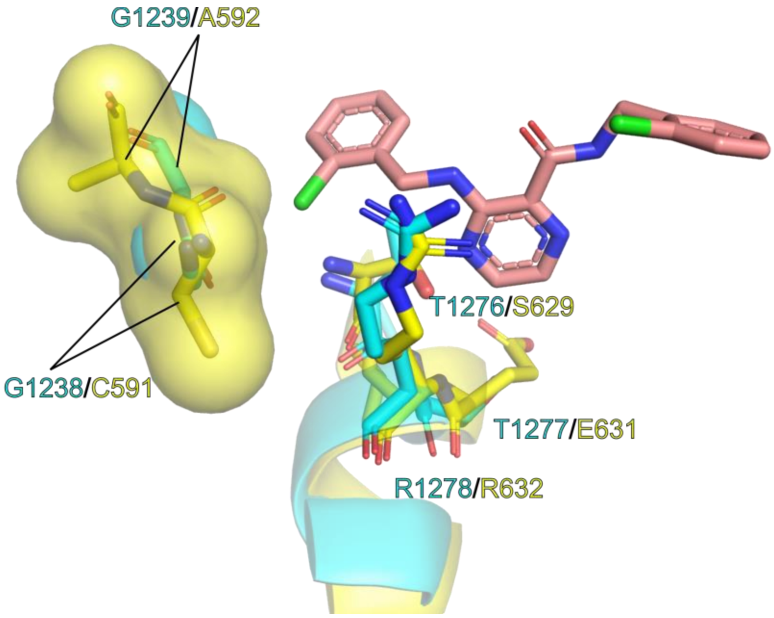

Figure S9. Superposition of the N-terminus of motif-3 α-helix of HcProRS:Pro:**3b** (cyan) and human ThrRS (yellow; PDB ID: 4HWT). The equivalent residues of Gly1238 and Gly1239 in HcProRS (shown as sticks surrounded by cyan surface representation) are replaced by Cys591 and Ala592 (shown as sticks surrounded by yellow surface representation). The presence of the side chain of Cys591 clearly reduces the flexibility of backbone of this residue which may cause the potential clash between carbonyl oxygen of Cys591 and ortho-substituent on C3-amine benzyl ring.

Synthesis of 3-(cyclohexanecarboxamido)-*N*-(2,3-dihydro-1*H*-inden-2-yl)pyrazine-2-carboxamide (**2a**, Adachi ligand)

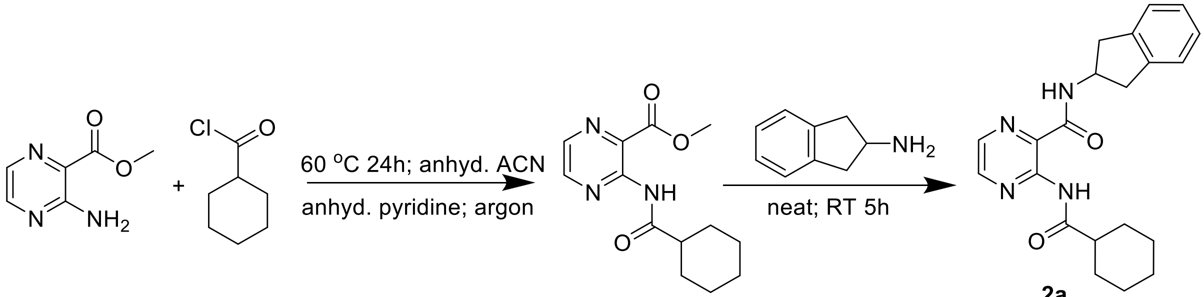

2a-0

2a
Adachi ligand

In a 100 mL dry round-bottomed flask filled with argon, 3.0 mmol of 3-aminopyrazine-2-carboxylic acid methyl ester (Fluorochem Ltd, Hadfield, UK) was partially dissolved in 20 mL of anhydrous acetonitrile (ACN) and stirred for 5 min. Then, 6 mmol (2.0 eq) of anhydrous pyridine was added and stirred for additional 5 min. To the reaction mixture, 5.4 mmol (1.8 eq) of cyclohexanoyl chloride was added dropwise, and the mixture was stirred at 60 ˚C for 24 h until the starting ester was completely consumed (as indicated by TLC, silica, hexane-EtOAc 1:1). The solvents were evaporated under vacuo, the crude was adsorbed on silica and purified using flash chromatography (silica, 0–40% EtOAc in hexane gradient elution). The product **2a-0** was isolated (60% yield) as a pale yellow viscous liquid, which solidified upon standing at room temperature.
To a 10 mL round-bottomed flask, 150 mg of methyl 3-(cyclohexanecarboxamido)pyrazine-2-carboxylate (**2a-0**) was mixed with 400 mg of 2-aminoindane (Sigma-Aldrich) and stirred for 5 h at room temperature neat (without any solvent). The crude mixture was purified using flash chromatography (silica, 0–55% EtOAc in hexane gradient elution). The product was isolated as a white solid with 97% yield.

3-(cyclohexanecarboxamido)pyrazine-2-carboxylate (**2a-0**). Light beige solid. Yield: 60%. mp 105.5–107.0 ˚C. Rf (Hex-EtOAc 1:1) = 0.30.

3-(cyclohexanecarboxamido)-N-(2,3-dihydro-1H-inden-2-yl)pyrazine-2-carboxamide (**2a**, Adachi ligand). White solid. Yield: 97%. mp 148.5–151.0 ˚C. R_f (Hex-EtOAc 1:1) = 0.33.
^{1}H NMR (500 MHz, $CDCl_3$) δ 11.98 (s, 1H, NHCO), 8.58 (d, *J* = 2.3 Hz, 1H, pyrazine), 8.33 (d, *J* = 8.1 Hz, 1H, CONH), 8.12 (d, *J* = 2.3 Hz, 1H, pyrazine), 7.29 – 7.27 (m, 2H, indane ArH), 7.24 – 7.20 (m, 2H, indane ArH), 4.94 – 4.85 (m, 1H, indane aliph.), 3.44 (dd, *J* = 16.1, 7.2 Hz, 2H, indane aliph.), 2.98 (dd, *J* = 16.1, 4.8 Hz, 2H, indane aliph.), 2.48 – 2.41 (m, 1H, cHex), 2.12 – 2.01 (m, 2H, cHex), 1.91 – 1.80 (m, 2H, cHex), 1.74 – 1.55 (m, 3H, cHex), 1.42 – 1.23 (m, 3H, cHex). ^{13}C NMR (126 MHz, $CDCl_3$) δ 174.53, 165.16, 149.59, 146.42, 140.42, 136.13, 129.01, 126.96, 124.81, 50.55, 47.36, 39.95, 29.34, 25.67, 25.61. MS ESI+ (MeOH:H_2O, 80/20, v/v) t = 200 °C; m/z 751 = $[2M+Na]^+$; 387 = $[M+Na]^+$

NMR spectra of **2a**

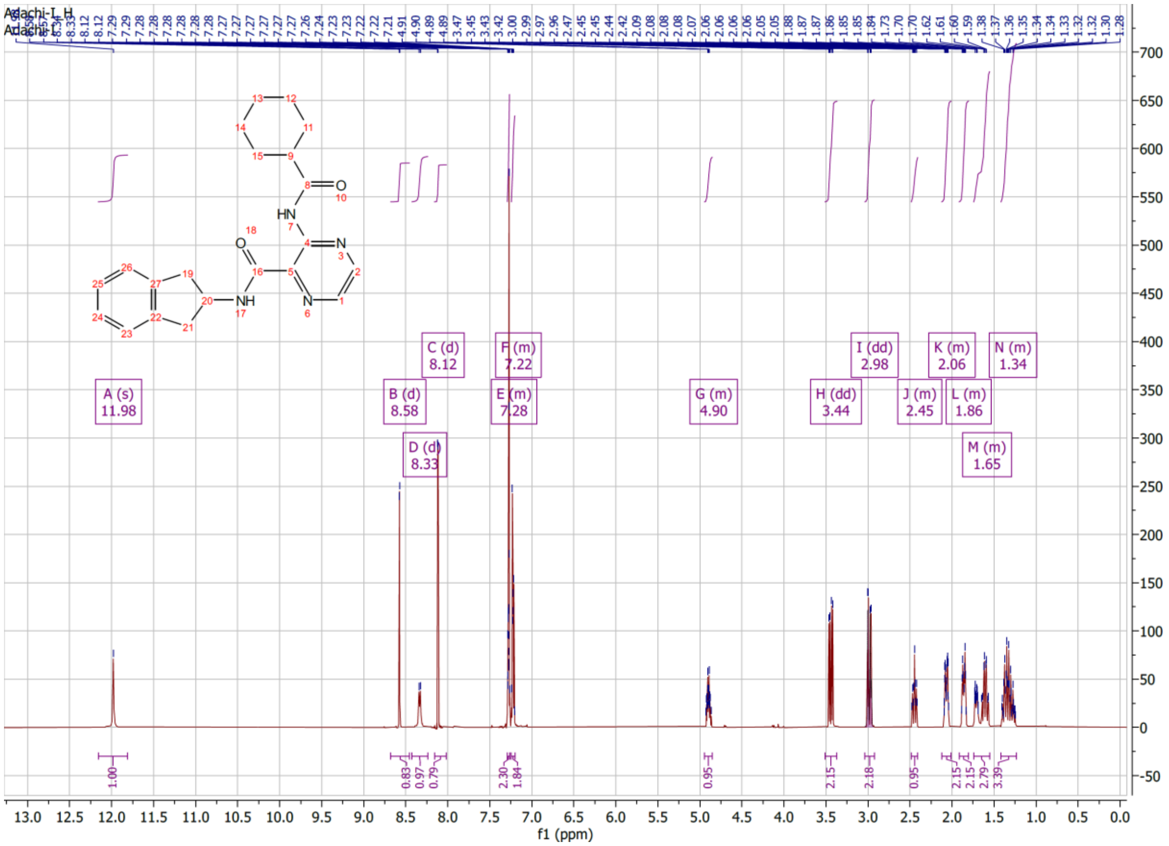

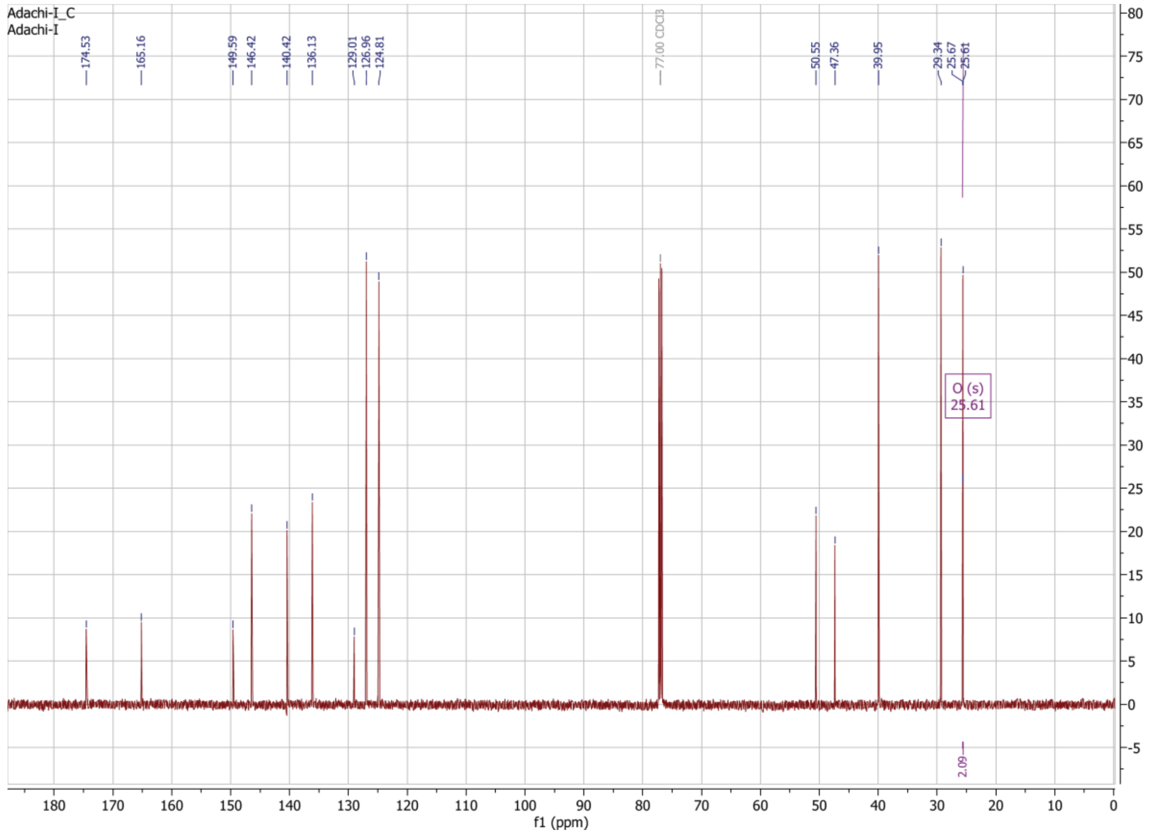

MS spectra of **2a**

ESI+ (MeOH:H_2O, 80/20, v/v) t = 200 °C; 751 = $[2M+Na]^+$; 387 = $[M+Na]^+$

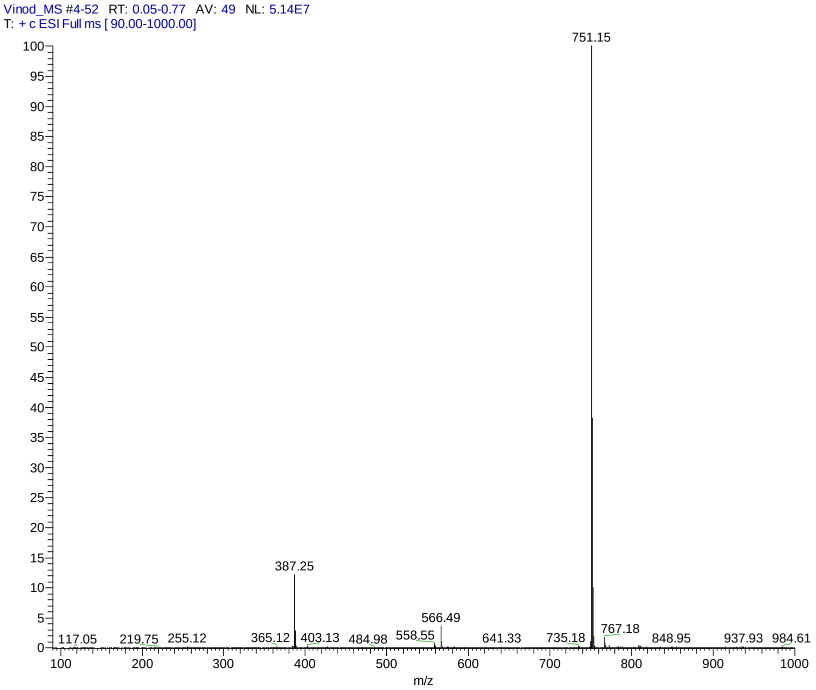

ESI+ (MeOH:H_2O, 80/20, v/v) t = 300 °C; 751 = $[2M+Na]^+$; 387 = $[M+Na]^+$; 767 = $[2M+K]^+$

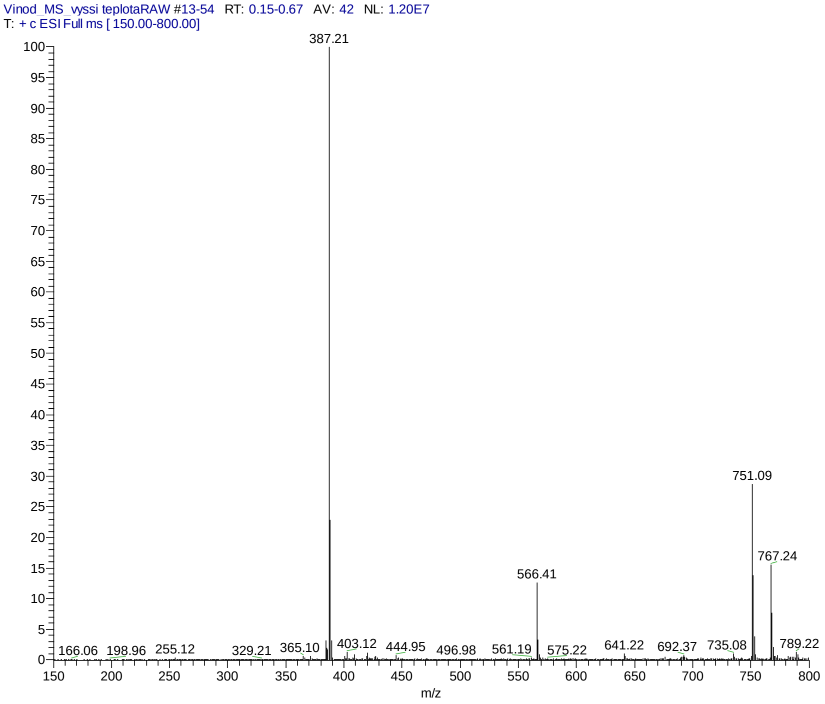

Supplement: Supplementary file 1 [file ijms-22-07793-s001.zip › ijms-1253758-supplementary.pdf]
